# Sonochemical degradation of CCl_4_ in an Ar-H_2_ dissolved aqueous solution^☆^

**DOI:** 10.1016/j.ultsonch.2026.108108

**Published:** 2026-10-10

**Authors:** Aerfate Abulikemu, Kenji Okitsu

**Affiliations:** Graduate School of Sustainable System Sciences, Osaka Metropolitan University, 1-1 Gakuen-cho, Naka-ku, Sakai, Osaka 599-8531, Japan

**Keywords:** CCl_4_, Sonochemical degradation, Ar-H_2_ mixed-gas system, ^•^H radicals, Bubble temperatures, Bubble number, Negative effects of H_2_

## Abstract

The sonochemical degradation of CCl_4_ in an Ar-H_2_ dissolved aqueous solution was investigated. In particular, the proportion of dissolved H_2_ in the total dissolved gas was analyzed as a key variable. The observed non-linear correlation between the degradation rate of CCl_4_ and H_2_ proportion in the Ar-H_2_ mixed gas system suggested two opposing effects of H_2_ on the system. As the positive effect of H_2_, the increase in the concentration of ^•^H radicals formed from H_2_ could contribute to the reductive degradation of CCl_4_. On the other hand, as the negative effects of H_2_, the decreases in bubble number and bubble temperatures could suppress the formation of ^•^H radicals and the degradation of CCl_4_. This interpretation was supported by the multiple experimental results of sonoluminescence (SL) measurement, H_2_O_2_ formation, quantitative evaluation of dissolved gas concentration, and analysis of bubble temperature and carbon-containing products in the Ar-H_2_ mixed gas system.

## 1 Introduction

Ultrasonic water treatment technology via acoustic cavitation can be considered a promising environmentally friendly method for removing pollutants from water. Acoustic cavitation is a phenomenon involving the formation, growth, and collapse of microbubbles in an aqueous solution. The adiabatic collapse of the cavitation bubbles generates extreme and transient conditions of high temperature and high pressure [1], [2], [3]. At such high temperatures, water molecules thermally degrade into ^•^H radicals and ^•^OH radicals, which can be used for the degradation of organic pollutants in water [4], [5]. In general, the dissolved gas is origin of bubble nuclei in an aqueous solution. Therefore, the type of the dissolved gas significantly affects bubble number, bubble size, bubble dynamics, bubble conditions such as temperature and pressure, and radical production [6], [7]. For example, Eqs. (1), (2) indicate that the specific heat ratio (γ) of the dissolved gas inside a cavitation bubble determines the theoretical maximum temperature (*T_max_*) and pressure (*P_max_*) reached by the collapse of a bubble, where *T_0_* and *P_0_* represent the initial temperature of the bulk solution and the pressure inside the bubble, respectively [8], [9].

(1) $$T_{\max}=\frac{T_{0}P_{\max}\left(\gamma -1\right)}{P_{0}}$$

(2) $$P_{\max}={P_{0}\left(\frac{P_{\max}\left(\gamma -1\right)}{P_{0}}\right)}^{\gamma /(\gamma -1)}$$

Carbon tetrachloride (CCl_4_) is known as a non-flammable, colorless, volatile organic compound (VOC). It was widely used in fire extinguishers, refrigerators, and dry-cleaning agents until its potential damage to human liver, kidneys and nervous system was recognized [10], [11]. However, global CCl_4_ emissions remain substantially higher than expected, despite its elimination from most industrial applications. An *et al*. reported that China accounted for approximately half of global CCl_4_ emissions during 2011–2020, with 4–15 Gg/year of the emissions remaining unexplained [12]. Li *et al*. indicated that the manufactures of chemical products, raw chemical materials, and general-purpose machinery are the main potential industrial source of CCl_4_ emissions in eastern China during 2021–2022 due to the high concentration of CCl_4_ in the waste gas [13]. In addition to entering groundwater through improperly treated industrial wastewater, CCl_4_ can infiltrate into groundwater through rainfall, soil adsorption, and other environmental processes due to its long atmospheric lifetime [14], [15], [16]. The persistent pollution caused by CCl_4_ and its strong resistance to natural degradation warrant further attention from researchers. Since Weissler *et al*. reported in 1950 that CCl_4_ can be effectively degraded to oxidizing chlorine in aqueous solution by ultrasonic irradiation, sonochemical degradation of CCl_4_ has been investigated significantly over the past decades [17], [18], [19], [20], [21]. It is suggested that CCl_4_ is mainly degraded by the high temperatures generated during cavitation, and the degradation process does not involve ^•^OH radicals in the aqueous solution [22], [23].

In our previous study, a pretreatment solution (alcohol-CCl_4_) was first used to investigate the sonochemical degradation of CCl_4_ in aqueous solution [24]. In the results, the solution homogeneity and the formation of ^•^H radicals were regarded as positive effects of alcohol on the degradation of CCl_4_ under Ar. In this study, to further enhance the degradation of CCl_4_, the sonochemical degradation of CCl_4_ in an Ar-H_2_ mixed gas system was investigated. We also presented a detailed investigation to evaluate the effect of H_2_ on the acoustic cavitation of Ar-H_2_ mixed gas system.

## 2 Experimental

### 2.1 Material and apparatus

In this study, high-purity reagents were used without extra purification. They included carbon tetrachloride (CCl_4_, 99.8 %, Wako), ethylene glycol (EG, 99.5 %, Wako), Na_2_CO_3_ (99.8 %, Wako), NaHCO_3_ (99.5 %, Wako), methanol (MeOH, 99.8 %, Fujifilm Wako), H_2_SO_4_ (95.0 %, Fujifilm Wako), hydrogen tetrachloroaurate(Ⅲ) tetrahydrate (HAuCl_4_·4H_2_O, 99.0 %, Fujifilm Wako), NaBr (99.5 %, Fujifilm Wako), *t-*butyl alcohol (*t-*BuOH, 99.0 %, Wako), potassium hydrogen phthalate (KHC_8_H_4_O_4_, 99.9 %, Fujifilm Wako), KI (99.5 %, Fujifilm Wako), NaOH (97.0 %, Wako), and hexaammonium heptamolybdate tetrahydrate ((NH_4_)_6_Mo_7_O_24_·4H_2_O, 99.0 %, Fujifilm Wako). All aqueous solutions were prepared using water purified with a Millipore Direct-Q 3UV system.

All experiments were conducted in a 20.0 ± 0.1 ℃ water bath maintained by a constant temperature circulator (CTP-1000, EYELA). All solutions were irradiated with 200 kHz ultrasound using an ultrasonic transducer (type 4611, Kaijo, Lot. No. 00102) and an ultrasonic generator (type 4021, Kaijo, Lot. No. 11EA00103) at a power dial setting of 8, where the calorimetric power was measured as 13.6 W. A short irradiation time of 5 min was chosen to minimize potential effects such as degassing, changes in gas composition, changes in chemical components, and changes in bulk solution temperature, although the water bath temperature was precisely controlled. A cylindrical glass reaction vessel and another with a quartz window for sonoluminescence (SL) measurement were used in this study. To reduce acoustic reflection during the experiments, the reaction vessels were placed 4.0 mm above the surface of ultrasonic transducer. Each experiment was repeated at least three times under each condition.

### 2.2 Ar-H_2_ mixed gas system

Each solution was placed in the reaction vessel and bubbled with an Ar-H_2_ gas mixture for 30 min in a 20.0 ± 0.1 ℃ water bath. The gas flow rates of H_2_ and Ar during bubbling were controlled as follows: H_2_/Ar = 0/100, 10/90, 20/80, 30/70, 40/60 (mL/min). The relationship between the ratio of H_2_/Ar flow rate and the proportion of H_2_ in the Ar-H_2_ dissolved solution is shown in Fig. S1 in Supplementary Data. After bubbling, the solution was left to stand for another 30 min to ensure the dissolution equilibrium of the mixed gas. Before ultrasonic irradiation, the amounts of Ar and H_2_ in the gas phase were analyzed using a gas chromatograph (GC-2014AT, TCD model, Shimadzu). The GC-TCD analysis conditions were as follows: the carrier gas was N_2_ at a flow rate of 30 mL/min, the separation column was Molecular Sieve 5A, the column temperature was 70 ℃, the detector temperature was 150 ℃, the injector temperature was 120 ℃, and the sample injection amount was 0.50 mL.

In our system, the actual concentrations of dissolved Ar and H_2_ in the solution (*c*, M) were determined from their partial pressure (*P*, bar) using Henry’s law as shown in Eq. (3), where *k_H_* is the Henry’s law constant. Under the condition of this study (293.15 K, 1 bar), the Henry’s law constants (M/bar) of Ar and H_2_ are 1.5 × 10^-3^ and 8.0 × 10^-4^, respectively [25].

(3) $$c=k_{H}\times P$$

### 2.3 Degradation of CCl_4_

A certain volume of CCl_4_ was pre-dissolved in EG to prepare a pretreatment solution (EG-CCl_4_). An Ar-H_2_ gas mixture was dissolved in 100 mL of pure water using the method described in Section 2.2. The EG-CCl_4_ pretreatment solution was added to the solution to prepare an Ar-H_2_ dissolved aqueous solution of CCl_4_, where the initial concentration of CCl_4_ was 1.0 mM. Samples were taken before and after 5 min ultrasonic irradiation. The initial degradation rate of CCl_4_ obtained during the first 5 min of ultrasonic irradiation was used for data analysis.

Since CCl_4_ is volatile and ultrasonic irradiation causes degassing, the degradation progress could not be evaluated based on the CCl_4_ concentration in the solution. In addition, it has been reported that the other major intermediates, such as C_2_Cl_4_ and C_2_Cl_6_, were detected in trace amounts and were further degraded during subsequent sonication [19]. Therefore, the degradation of CCl_4_ was evaluated by analyzing the amount of Cl^-^, the final major product formed during the entire sonication process. The concentration of Cl^-^ was measured using an ion chromatograph (761 compact IC, Metrohm) with a column heater (U-620, Sugai Chem). The IC analysis conditions were as follows: the separation column was Shodex IC SI-90 4E, the column temperature was 40 ℃, the regeneration solution was an aqueous solution of H_2_SO_4_ (40 mM), the eluent was a mixture of aqueous solutions of NaHCO_3_ (1.7 mM), Na_2_CO_3_ (1.8 mM), and 5 vol% of methanol at a flow rate of 1.0 mL/min.

### 2.4 Analysis of sonoluminescence (SL)

An Ar-H_2_ gas mixture was dissolved in 100 mL of pure water using the method described in Section 2.2. The SL spectrum generated during sonication was measured from 290 to 600 nm using a modified fluorescence spectrophotometer (RF-6000, Shimadzu), where both the water bath and the reaction vessel were equipped with quartz windows and were placed in the sample chamber. The background spectrum obtained without ultrasonic irradiation was subtracted from each SL spectrum.

### 2.5 Formation of H_2_O_2_

An Ar-H_2_ gas mixture was dissolved in 100 mL of pure water using the method described in Section 2.2. Samples were taken before and after 5 min ultrasonic irradiation. The concentration of H_2_O_2_ was determined by the KI colorimetric method using a UV–visible spectrophotometer (Shimadzu, UV-2450). Solutions A and B were added to samples and the oxidization of I^-^ by H_2_O_2_ was evaluated from the absorption peak of I_3_^-^ at 352 nm, where Solution A was an aqueous solution of KHC_8_H_4_O_4_ (0.10 M) and Solution B was an aqueous solution including NaOH (0.05 M), KI (0.40 M), and (NH_4_)_6_Mo_7_O_24_ (1.6 × 10^-4^ M) [26]. The concentration of H_2_O_2_ was determined using the molar absorptivity of I_3_^-^ (2.6 × 10^4^ M^−1^ cm^−1^). The formation rate of H_2_O_2_ obtained during the first 5 min of ultrasonic irradiation was used for data analysis.

### 2.6 Measurement of bubble temperature

An Ar-H_2_ gas mixture was dissolved in 100 mL of pure water using the method described in Section 2.2. *t-*BuOH was added to obtain an initial concentration of 20 mM. The volume of the gas phase in the reaction vessel was 109 mL. Gaseous samples were taken before and after 5 min ultrasonic irradiation and measured using a gas chromatograph (GC-2014AT, FID model, Shimadzu) equipped with a methanizer (MT221, GL Sciences). The GC-FID analysis conditions were as follows: the carrier gas was N_2_ at a flow rate of 40 mL/min, H_2_ was at a flow rate of 60 mL/min, air was at a flow rate of 30 mL/min, the methanizer temperature was 350 ℃, the separation column was Shincarbon ST, the column temperature was 150 ℃, the detector temperature was 200 ℃, the injector temperature was 170 ℃, and the sample injection amount was 0.50 mL. The formation rate of carbon-containing products obtained during the first 5 min of ultrasonic irradiation was used for data analysis.

## 3 Results and discussion

The effect of dissolved H_2_ on the sonochemical degradation of CCl_4_ in aqueous solution was investigated. A pretreatment solution (EG-CCl_4_) was used in order to eliminate the effect of solution homogeneity. The effects of EG were discussed in our previous work [24]. Fig. 1 shows the degradation rate of CCl_4_ as a function of H_2_ proportion in an Ar-H_2_ dissolved aqueous solution during 5 min of sonication, where H_2_ proportion was measured as described in Section 2.2. The results at H_2_ proportions below 30 % are primarily considered due to the dominant negative effect of H_2_ on the system at higher proportions, as discussed later. In Fig. 1, with increasing H_2_ proportion in an Ar-H_2_ dissolved aqueous solution, the rate of the degradation of CCl_4_ was accelerated and reached a peak at the H_2_ proportion of 5.9 %. The rate then decreased as the proportion of H_2_ further increased. This non-linear relationship indicates a competition between two opposing effects of dissolved H_2_ on the degradation of CCl_4_.

**Fig. 1:**
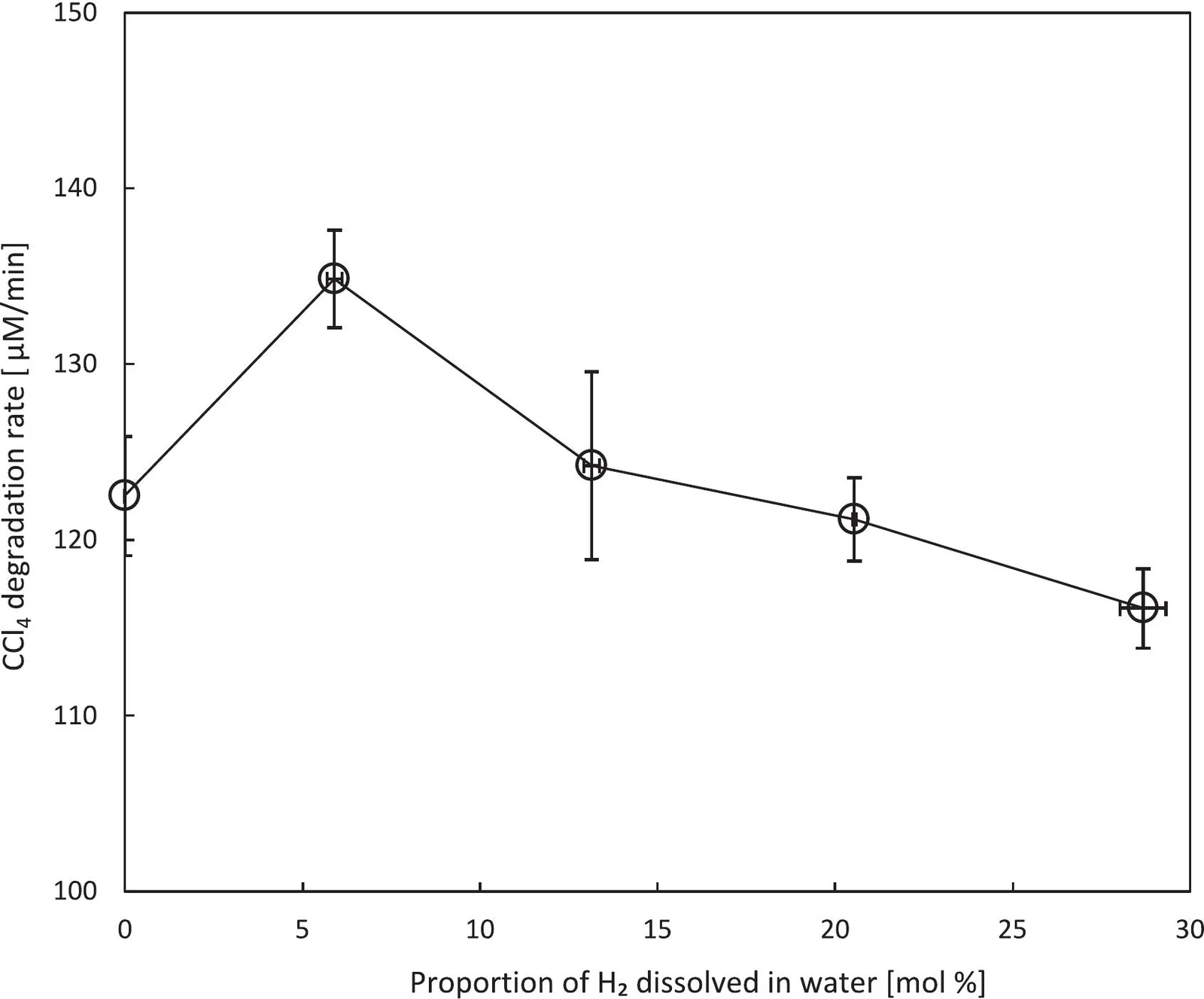
Degradation rate of CCl_4_ in an Ar-H_2_ dissolved aqueous solution, [CCl_4_]_0_ = 1.0 mM, [EG]_0_ = 61 mM.

It is believed that dissolved H_2_ reacts with ^•^OH radicals formed from sonolysis of water, as shown in Eqs. (4), (5) [27]. In addition, ^•^H radicals can be generated from dissolved H_2_ under ultrasonic irradiation as shown in Eq. (6) [28]. Consequently, the higher concentration of ^•^H radicals in the presence of H_2_ could lead to the enhancement of the degradation of CCl_4_ in aqueous solution.

(4) $$H_{2}O\;\overset{\;)))}{\to }\;∙H+\;∙OH$$

(5) $$H_{2}+\;∙OH\to \;∙H+H_{2}O$$

(6) $$H_{2}\;\overset{\;)))}{\to }\;2∙H$$

The sonochemical formation of ^•^H radicals in an Ar-H_2_ mixed gas system has been investigated by Gutiérrez *et al*. at the frequency of 1 MHz [29]. Under Ar-H_2_ gas mixtures, the reduction rates of inorganic ^•^H radical scavengers such as Au(III), MnO_4_^-^, Ag^+^, etc. reached a maximum at about H_2_ volume fraction of 20 %. Their results support the discussion above that the formation of ^•^H radicals is enhanced in an Ar-H_2_ mixed gas system compared with that in a pure Ar gas system. However, in our results for the sonication of an aqueous Au(Ⅲ) solution containing dissolved Ar-H_2_, as shown in Figs. S2 and S3 in Supplementary Data, the reduction rate of Au(Ⅲ) did not change significantly with the H_2_ proportion due to extremely low scavenging efficiency of Au(Ⅲ) toward ^•^H radicals. On the other hand, it is suggested that the introduction of H_2_ into an Ar-saturated aqueous solution has a negative effect on the system, and this effect increases as the H_2_ proportion increases. Therefore, the formation of ^•^H radicals would be suppressed at high H_2_ proportions, whereas it would not be significantly affected at lower proportions.

The negative effects of H_2_ on an Ar-H_2_ mixed gas system was evaluated. Pure water in the absence of EG was used in this section to simplify the system. Fig. 2 shows SL intensity in an Ar-H_2_ dissolved aqueous solution relative to that under pure Ar conditions as a function of H_2_ proportion. As shown in Fig. 2, with an increase in the proportion of dissolved H_2_, quenching of the SL emission occurred. This result agrees well with MacIntyre’s study, which reported that the SL of H_2_ is significantly lower than that of Ar [30]. It is well known that water saturated with noble gases generally exhibits stronger SL than polyatomic gases due to a high γ value, a low thermal conductivity, and low energy loss [31]. In the present Ar-H_2_ mixed gas system, the observed SL quenching was explained from the following aspects: (1) decrease in the amount of ^•^OH radicals, (2) decrease in the number of bubbles, and (3) decrease in the bubble temperatures.

**Fig. 2:**
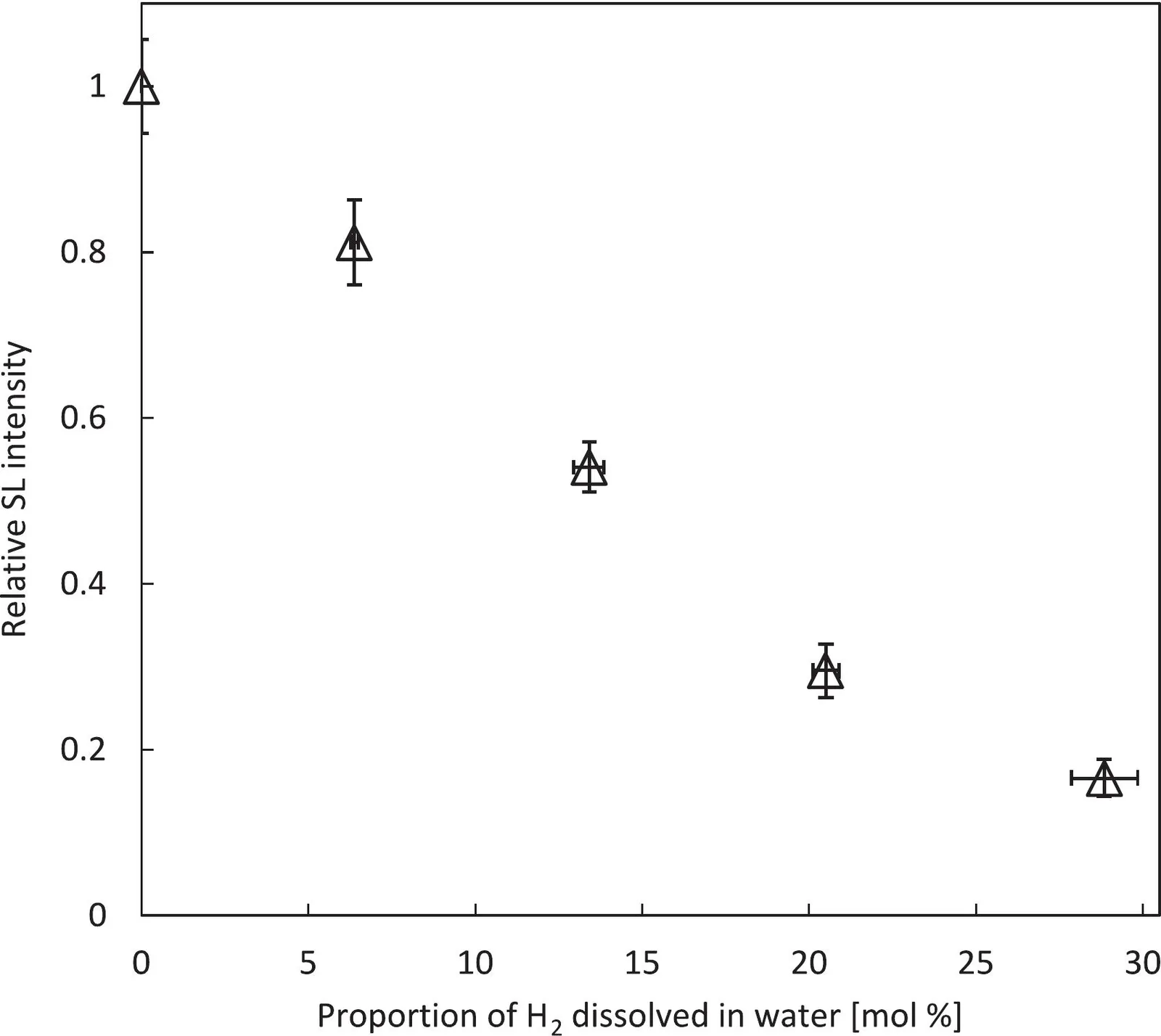
Relative SL intensity at different H_2_ proportion in an Ar-H_2_ dissolved aqueous solution.

As shown in Fig. S4 in Supplementary Data, one of the most notable changes in the SL spectrum was the quenching of the ^•^OH radical emission at 310 nm [32]. The SL intensity at 310 nm, associated with ^•^OH radical formation, decreased as the H_2_ proportion increased. This result suggests that H_2_ and ^•^H radicals in an aqueous solution could scavenge ^•^OH radicals formed from sonolysis of water. Fig. 3 shows the formation rate of H_2_O_2_ in the Ar-H_2_ dissolved aqueous solution as a function of H_2_ proportion. The result indicates that the yield of H_2_O_2_ formed from ^•^OH radicals decreased with an increase in H_2_ proportion, which is also consistent with the discussion above.

**Fig. 3:**
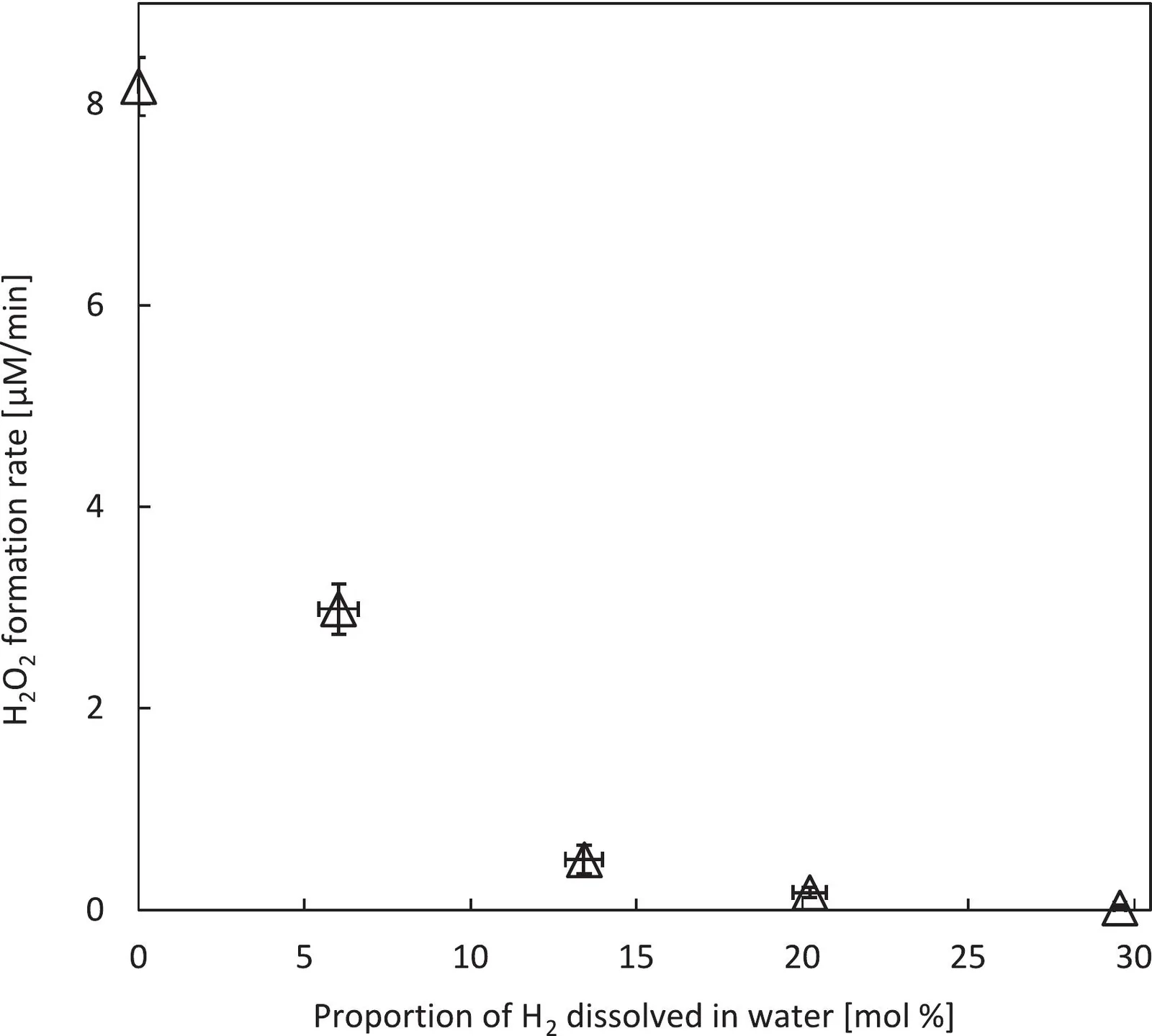
Formation rate of H_2_O_2_ from sonolysis of an Ar-H_2_ dissolved aqueous solution.

The introduction of H_2_ into an Ar-saturated system would have a negative effect on the number of the bubbles. Based on the Henry’s law constants mentioned above, the solubility of Ar in water is approximately twice as high as that of H_2_. With an increase in the H_2_ proportion in an Ar-H_2_ dissolved aqueous solution, the total amount of dissolved gas in the solution decreases. For example, the sum concentration of H_2_ and Ar dissolved gas estimated at each H_2_ proportion in Fig. 1 is as follows: 1.52 mM (H_2_ proportion: 0 %), 1.44 mM (5.9 %), 1.37 mM (13.1 %), 1.31 mM (20.5 %), and 1.28 mM (28.7 %). A lower amount of dissolved gas would reduce the number of active bubbles in an aqueous solution [33], [34].

The effect of H_2_ on the bubble temperatures in the Ar-H_2_ mixed gas system was investigated. The bubble temperatures during cavitation were estimated by the methyl radical recombination (MRR) method [35]. In this method, as shown in Eq. (7), the rate constants for ethylene (C_2_H_4_) formation (k_1_) and ethane (C_2_H_6_) formation (k_2_) exhibit different sensitivities to temperature, where k_1_ = 1.0 × 10^16^ exp (−134 kJ/RT) [M^−1^ s^−1^], and k_2_ = 2.4 × 10^14^ T^−0.4^ [M^−1^ s^−1^]. The ratio of k_1_ to k_2_ equals to the ratio of the yield of C_2_H_4_ plus acetylene (C_2_H_2_) (Y(C_2_H_4_) + Y(C_2_H_2_)) to the yield of C_2_H_6_ (Y(C_2_H_6_)), where C_2_H_2_ is assumed to be formed from C_2_H_4_. Thus, the bubble temperatures can be estimated by measuring the amounts of C_2_ hydrocarbons [36]. Since the sonochemical degradation of EG does not readily proceed and the amounts of C_2_ hydrocarbons generated is insufficient for the analysis as shown in Fig. S5 in Supplementary Data, *t-*BuOH was used instead of EG in this section.

(7) $$\frac{Y\left(C_{2}H_{4}\right)+Y\left(C_{2}H_{2}\right)}{Y(C_{2}H_{6})}=\frac{k_{1}}{k_{2}}$$

Fig. 4 shows the estimated bubble temperatures and the formation rate of carbon-containing products (CO, CO_2_, CH_4_, C_2_H_2_, C_2_H_4_, C_2_H_6_) as a function of H_2_ proportion during 5 min sonication of an Ar-H_2_ dissolved aqueous solution of *t-*BuOH. The temperatures estimated under a pure Ar atmosphere were consistent with recent numerical simulations predicting temperatures of approximately 3000–4500 K at frequencies around 200 kHz. [37], [38]. As shown in Fig. 4, the estimated bubble temperatures decreased with increasing H_2_ proportion in the Ar-H_2_ mixed gas system. The formation of C_2_H_4_ and C_2_H_2_ might be inhibited by the presence of H_2_, resulting in an estimation error. However, the bubble temperatures are still believed to decrease according to the decrease in the yields of carbon-containing products [39]. These results support the theory that due to the lower γ value of H_2_ (1.41 at 15 ℃) than that of Ar (1.67 at 15 ℃), a cavitation bubble filled with H_2_ or Ar-H_2_ generates a lower temperature than an Ar-filled bubble does upon collapse [40], [41]. In addition, with increasing H_2_ proportion in the Ar-H_2_ system, only slight decreases in the estimated bubble temperatures were observed while the SL emissions were quenched extremely as shown in Fig. 2. It is believed that in the presence of low proportion of dissolved H_2_, the estimated bubble temperatures by MRR method, regarded as the average bubble temperatures over time and volume, are not as significantly affected as the peak bubble temperatures indicated by SL [42], [43]. The observed SL quenching is the result of multiple effects of H_2_ on the system as described above.

**Fig. 4:**
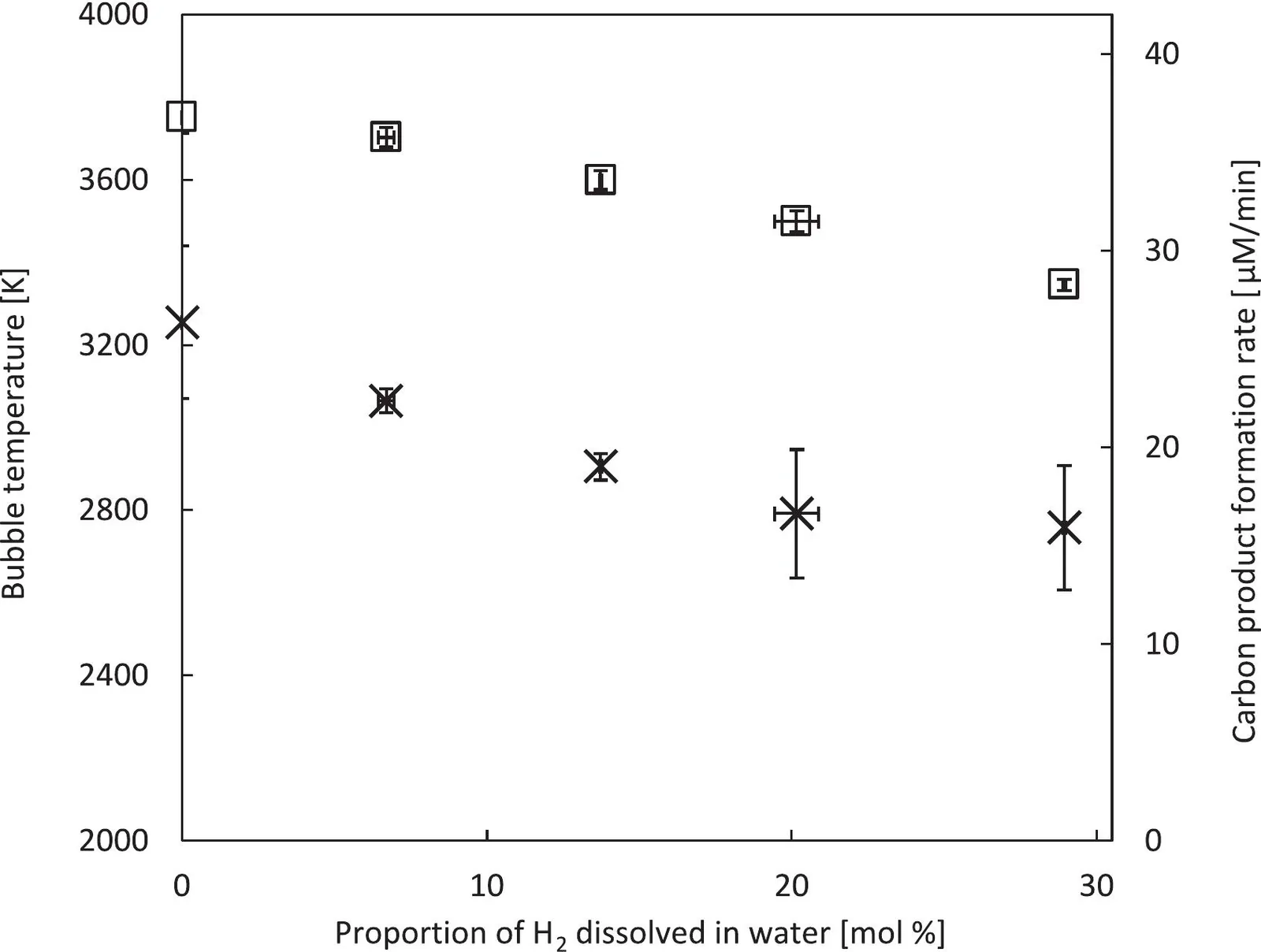
Estimated bubble temperatures [□], and formation rate of carbon-containing products [✕] in an Ar-H_2_ dissolved aqueous solution of *t-*BuOH, [*t-*BuOH]_0_ = 20 mM.

It is considered that highly volatile CCl_4_ tends to enter the interior of cavitation bubbles and undergoes rapid thermal degradation at high temperatures inside the bubbles [22]. CCl_4_ is also present at the interface region of the cavitation bubbles due to its hydrophobicity. In this region, CCl_4_ would undergo thermal degradation under sustained high temperatures and reductive degradation in the presence of high concentrations of ^•^H radicals. In contrast, non-volatile and hydrophilic ^•^H radical scavengers, such as Au(Ⅲ) ions, distributed throughout the solution, could react with ^•^H radicals at the interface region of the cavitation bubbles or with ^•^H radicals that diffuse into the bulk solution. Consequently, the rate of Au(Ⅲ) reduction by ^•^H radicals was substantially lower than the rate of CCl_4_ degradation. A more ideal indicator for analyzing the ^•^H radical dynamics in the CCl_4_ system remains to be identified in future studies.

In the Ar-H_2_ system, the formation of ^•^H radicals competed with the negative effects of H_2_ as H_2_ proportion increased. Therefore, the enhancement of the sonochemical degradation of CCl_4_ was only observed at H_2_ proportions below 13.1 %. The enhanced reductive degradation of CCl_4_ at high concentration of ^•^H radicals could offset the weakening of the thermal degradation of CCl_4_ caused by decreased bubble temperatures and a reduced number of bubbles. 5.9 % of H_2_ could be an optimum proportion in the Ar-H_2_ mixed gas system for degradation of CCl_4_, where the two opposing effects achieved the desired balance.

In addition, due to the limited reductive effect of ^•^H radicals, introduction of a small amount of H_2_ into a pure Ar system may not result in a proportional enhancement in the CCl_4_ degradation rate, with the observed enhancement being only around 12 %. However, the findings of this study are expected to be applicable to other halocarbons that can react with ^•^H radicals. Recently, anaerobic reductive dechlorination has attracted considerable attention as a method for the degradation of organohalogen compounds [44], [45]. In an ultrasonic system, ultrasonic irradiation has been reported to activate iron powder and promote the reductive dechlorination of CCl_4_ under anaerobic conditions [46]. The findings obtained in the Ar-H_2_ mixed gas system suggest that such conditions may provide a favorable environment for the reductive degradation of organohalogen compounds and may contribute to the development of advanced reductive processes for water treatment in the future.

## 4 Conclusion

The sonochemical degradation of CCl_4_ in an aqueous solution was investigated in an Ar-H_2_ mixed gas system. The degradation of CCl_4_ was found to be determined by the competing positive and negative effects of H_2_ in the system. The degradation rate of CCl_4_ increased and reached a maximum at the H_2_ proportion of 5.9 %. This would be attributed to the increasing concentration of ^•^H radicals in the presence of H_2_. However, with a further increase in the H_2_ proportion, the negative effect of H_2_ on the system became dominant, resulting in a decrease in the degradation rate of CCl_4_. The result of SL indicated that the negative effect of H_2_ increased with an increase in H_2_ proportion. It was also confirmed that the increasing proportion of H_2_ in the system would result in decreases in the H_2_O_2_ formation rate, the number of bubbles, and the bubble temperatures. The findings of this study may provide insights into the sonochemical degradation of other organic pollutants that are susceptible to reaction with ^•^H radicals.

## CRediT authorship contribution statement

**Aerfate Abulikemu:** Writing – review & editing, Writing – original draft, Investigation, Conceptualization. **Kenji Okitsu:** Writing – review & editing, Supervision, Investigation, Funding acquisition, Conceptualization.

## Declaration of competing interest

The authors declare that they have no known competing financial interests or personal relationships that could have appeared to influence the work reported in this paper.
