# Supplementary material for: Sonochemical degradation of CCl4 in an Ar-H2 dissolved aqueous solution
Source: Ultrason Sonochem. 2026 Oct 10;134:108108. doi: 10.1016/j.ultsonch.2026.108108 (PMC13696826; doi:10.1016/j.ultsonch.2026.108108)
Supplement: Supplementary file 1 [file mmc1.docx]

**Supplementary Data**

Sonochemical degradation of CCl_4_ in an Ar-H_2_ dissolved aqueous solution

Aerfate Abulikemu, Kenji Okitsu^*^

Graduate School of Sustainable System Sciences, Osaka Metropolitan University,

1-1 Gakuen-cho, Naka-ku, Sakai, Osaka 599-8531, Japan.

*Corresponding author: Kenji Okitsu

Tel. & Fax: +81-72-254-9506, E-mail: okitsu@omu.ac.jp

**1. Controlling the proportion of dissolved H_2_ in water by varying the ratio of H_2_/Ar flow rate**

**Fig. S1.** Relationship between the ratio of H_2_/Ar flow rate and the proportion of H_2_ in an Ar-H_2_ dissolved aqueous solution.

**2. Reduction of Au(Ⅲ)**

An Ar-H_2_ gas mixture was dissolved in 0.2 mM of a HAuCl_4_ aqueous solution using the method described in Section 2.2. The concentration of Au(Ⅲ) was measured using a UV–visible spectrophotometer (Shimadzu, UV-2450). A NaBr-saturated solution (0.20 mL) was added to each sample solution (2.0 mL) to monitor the absorption peak corresponding to [AuBr_4_]^-^ at 380 nm. The rate of Au(Ⅲ) reduction was measured by analyzing the change in concentration during 10 min sonication. It was confirmed that the rate did not change with the H_2_ proportion as expected, as shown in Figs. S2 and S3. Considering the extremely low scavenging efficiency of Au(Ⅲ) toward •H radicals, we considered that Au(Ⅲ) may not be an ideal indicator in this study.

**Fig. S2.** Reduction of Au(Ⅲ) in an Ar-H_2_ dissolved aqueous solution, [HAuCl_4_]_0_ = 0.2 mM.

**Fig. S3.** Reduction rate of Au(Ⅲ) in an Ar-H_2_ dissolved aqueous solution, [HAuCl_4_]_0_ = 0.2 mM, irradiation time: 10 min.

**3. SL spectra from Ar-H_2_ dissolved aqueous solutions at different H_2_ proportions**

**Fig. S4.** SL spectra from Ar-H_2_ dissolved aqueous solution at different H_2_ proportion, from top to bottom: 0, 6.4, 13.4, 20.5 and 28.8 mol %. The spectrum presented was selected as the one closest to the average SL intensity of three independent experiments. The background spectrum obtained without ultrasonic irradiation was subtracted from each SL spectrum.

**4. Carbon-containing products from sonication of EG aqueous solution**

**Fig. S5.** Yields of carbon-containing products from sonication of 60 mM EG aqueous solution under Ar. The products, from top to bottom, are: CO [○], CO_2_ [□], CH_4_ (△), and C_2_H_2_ (◇).

**5. Part of the original measurement data**

**5.1 Degradation of CCl_4_, part of the original data measured by the IC.**

Because the eluent needed to be freshly prepared each time, the calibration curve relating peak area to Cl^-^ ion concentration was re-established for each newly prepared eluent. No peak was observed before irradiation, so the value was assumed to be zero and the data were excluded from the analysis. A representative ion chromatogram of Cl^-^ is shown in Fig. S6.

**Table S1.** Cl^-^ data measured by IC as a function of H_2_ proportion.

| H_2_ proportion /mol % | Peak area of Cl^-^ ion | Concentration of Cl^-^ /μM |
| --- | --- | --- |
| 0 | 1199 | 2430 |
| 5.97 | 1275 | 2740 |
| 12.9 | 1151 | 2480 |
| 20.6 | 1100 | 2430 |
| 28.1 | 1040 | 2310 |

**Fig. S6.** Ion chromatogram of Cl^-^ formed from sonolysis of an Ar-H_2_ dissolved aqueous solution. Conditions: H_2_ proportion: 5.97 %, irradiation time: 5 min.

Retention time of Cl^-^: 4.83 min.

**5.2 Formation of H_2_O_2_, part of the original data measured by UV–visible spectrophotometer.**

No absorption was measured before irradiation, so the value was assumed to be zero and the data were excluded from the analysis. Before measurement, the samples were diluted fivefold by adding 2 mL solution A and 2 mL solution B to adjust the absorbance to within the measurable range of the spectrophotometer. The concentrations listed in the table were calculated by multiplying the measured concentrations by a dilution factor of 5. A representative UV-vis spectrum of H_2_O_2_ is shown in Fig. S7.

**Table S2.** H_2_O_2_ data measured by UV–visible spectrophotometer as a function of H_2_ proportion.

| H_2_ proportion /mol % | Absorption | Concentration of H_2_O_2_ /μM |
| --- | --- | --- |
| 0 | 0.2099 | 40.4 |
| 6.29 | 0.0711 | 13.7 |
| 13.5 | 0.0126 | 2.42 |
| 19.6 | 0.0049 | 0.94 |
| 29.6 | 0.0013 | 0.25 |

**Fig. S7.** UV-vis spectrum of H_2_O_2_ formed from sonolysis of an Ar-H_2_ dissolved aqueous solution. Conditions: H_2_ proportion: 6.29 %, irradiation time: 5 min.

**5.3 Measurement of bubble temperature, part of the original data measured by GC-FID.**

No peak was observed before irradiation, so the value was assumed to be zero and the data were excluded from the analysis. A representative GC chromatogram of formed carbon-containing products is shown in Fig. S8.

**Table S3.** C_2_H_2_, C_2_H_4_, and C_2_H_6_ data measured by GC as a function of H_2_ proportion

| H_2_ proportion /mol % | Peak area of C_2_H_2_ | [C_2_H_2_] /μM | Peak area of C_2_H_4_ | [C_2_H_4_] /μM | Peak area of C_2_H_6_ | [C_2_H_6_] /μM |
| --- | --- | --- | --- | --- | --- | --- |
| 0 | 1479000 | 21.2 | 591700 | 5.51 | 201600 | 1.84 |
| 6.97 | 1446000 | 20.8 | 638100 | 5.95 | 207300 | 1.89 |
| 13.8 | 1045000 | 15.0 | 527200 | 4.91 | 172400 | 1.57 |
| 19.3 | 956200 | 13.7 | 567700 | 5.29 | 186000 | 1.70 |
| 28.9 | 600100 | 8.62 | 414100 | 3.86 | 156800 | 1.43 |

**Fig. S8.** GC chromatogram of formed carbon-containing products in the presence of 20 mM *t-*BuOH. Conditions: H_2_ proportion: 6.97 %, irradiation time: 5 min.

Retention time: 1.29 min (CO), 1.88 min (CH_4_), 2.90 min (CO_2_), 5.43 min (C_2_H_2_), 7.32 min (C_2_H_4_), 9.76 min (C_2_H_6_).
